# HER2-positive breast cancer cells expressing elevated FAM83A are sensitive to FAM83A loss

**DOI:** 10.1371/journal.pone.0176778

**Published:** 2017-05-02

**Authors:** Courtney A. Bartel, Mark W. Jackson

**Affiliations:** 1 Department of Pathology, Case Western Reserve University, Cleveland, Ohio, United States of America; 2 Case Comprehensive Cancer Center, Case Western Reserve University, Cleveland, Ohio, United States of America; University of South Alabama, UNITED STATES

## Abstract

HER2-positive breast cancer (HER2+ BC) is an aggressive subtype with a poor prognosis. Although the antibody trastuzumab, which targets the HER2 growth factor receptor, has improved survival rates, patients often present with *de novo* resistance or acquire resistance after an initial response. Identifying new ways to target HER2 signaling will be critical for overcoming trastuzumab resistance. FAM83A is a novel oncogene identified by its ability to confer resistance to EGFR therapies, a receptor closely related to HER2. Moreover, a prior study identified hyper-tyrosine phosphorylated FAM83A in trastuzumab-resistant HER2+ BC. Here, we find that FAM83A expression is elevated in 36% of HER2+ BC tumors. In a panel of HER2+ BC cell lines, FAM83A expression is significantly increased in the trastuzumab-resistant derivatives relative to parental controls. shRNA-mediated ablation of FAM83A in the panel of HER2+ BC cell lines suppresses HER2+ BC cell growth in both 2D and 3D cell cultures, elevates apoptosis markers, and suppresses PI3K signaling. Growth inhibition following FAM83A knock-down, however, was independent of trastuzumab sensitivity, suggesting that FAM83A is a key signaling component in HER2+ BCs that could serve as a novel therapeutic target in both trastuzumab-resistant and trastuzumab-sensitive cancers.

## Introduction

HER2 is a member of the ErbB family of transmembrane receptor tyrosine kinases, which also includes EGFR/ErbB1, ErbB3/HER3, and ErbB4/HER4. Ligand-mediated stimulation of ErbB receptors causes auto-phosphorylation of key tyrosine residues that serve as docking sites for downstream effector pathways, including the PI3K/AKT and MAPK (MEK/ERK) signaling cascades, among others [1–5]. These receptor-mediated signaling events are key in regulating normal cell function by promoting growth and survival, but, when disrupted can lead to cancer [6–9]. HER2+ BC is an aggressive subtype with a poor prognosis [10]. Approximately 20% of BCs are classified as HER2+, defined by the presence of HER2 gene amplification [11]. Trastuzumab is a monoclonal antibody that specifically targets HER2 by binding to the extracellular domain of HER2, resulting in disruption of HER2 downstream effector signaling and activation of immune killing via antibody-dependent cell-mediated cytotoxicity, among other mechanisms [12–16]. In early stage disease, treatment with trastuzumab is effective in 85% of the cases [17]. However, the majority of patients who initially respond show disease progression within a year [16]. In addition, in metastatic HER2+ BC, as many as 65% of patients present with *de novo* trastuzumab resistance [18–20]. These statistics indicate that both *de novo* and acquired resistance limit the effectiveness of trastuzumab treatment.

Trastuzumab resistance mechanisms are unfortunately quite heterogeneous, making development of therapies to combat resistance more challenging. For example, HER2 can heterodimerize with other ErbB family members (EGFR, HER3, and HER4) as well as other growth factor receptors (IGF-1R, c-Met) [14, 21–23]. While trastuzumab can effectively block HER2 homodimer formation, it is not as effective in blocking HER2 heterodimers [24]. Pertuzumab was designed to inhibit HER2 heterodimerization with other receptors, and has increased overall survival when co-administered with trastuzumab (56.5 months compared to 40.8 months) [25,26]. However, as HER+ BC is more frequent in young women under 39 years of age, a median survival of 56.5 months (or 4.7 years) remains a significant problem [25]. Additional modes of trastuzumab resistance include 1) hyperactivation of PI3K/AKT signaling (by mutation of PI3K, overexpression of AKT or PDK1, or loss of PTEN function), 2) alternative translational initiation or splicing, or cleavage of HER2, resulting in the production of a p95-HER2 fragment that is catalytically active in the cytoplasm, 3) elevated SRC activity, 4) increased MUC4 and CD44 (receptor for hyaluronan) expression, which can shield HER2 from trastuzumab, and IGF-1R activation [26–29]. In addition, over 120 proteins have been shown to interact with HER2 according to the BioGrid database [30], many of which have not been well described and could potentially play a role in trastuzumab resistance. Thus, although therapies like pertuzumab provide some hope to combat resistance, additional targets will be necessary as new resistance mechanisms continue to emerge.

One potential target is the novel oncogene FAM83A. Using two unique forward genetic screens, the Jackson and Bissell laboratories identified a novel family of oncogenes named FAM83A-H that are capable of driving human mammary epithelial cell (HMEC) transformation [31, 32]. FAM83A was identified by the Bissell laboratory in a cDNA library screen for genes that drive resistance to EGFR tyrosine kinase inhibitors (TKIs) in triple negative breast cancer (TNBC) [32]. Studies of signaling complexes also revealed interactions between FAM83A and key signaling effectors, such as CRAF and PI3K [32, 33]. shRNA-mediated knockdown of FAM83A also inhibits signaling through the MAPK and PI3K/Akt signaling pathways [32]. Although many have tried to target the MAPK and PI3K/AKT pathways, such therapies often have a narrow therapeutic window as these pathways are also crucial for signaling in normal cells [34, 35]. Importantly, FAM83A expression is elevated in breast cancers relative to normal breast tissue, and FAM83A protein is not expressed in host human tissues, suggesting it could have a wider therapeutic index [32, 33, 36]. Because of the known role FAM83A plays in promoting EGFR TKI therapeutic resistance and downstream signaling, we hypothesized that it may also confer resistance to other ErbB therapeutics, such as trastuzumab. This idea was further supported by a phospho-proteomics study that identified FAM83A as hyper-tyrosine phosphorylated in a trastuzumab-resistant HER2+ BC cell line [37]. Here, we show that FAM83A is overexpressed in HER2+ BCs and in trastuzumab-resistant breast cancer cell lines. Furthermore, knockdown of FAM83A expression severely inhibits proliferation and induces apoptosis in a panel of HER2+ BC cells. Thus, we propose that FAM83A could serve as a target for inhibiting the growth of all HER2+ BCs, including those that are resistant to trastuzumab.

## Materials and Methods

### FAM83A expression in HER2+ breast cancer

*FAM83A* expression was examined in HER2+ breast cancer using TCGA data TissueScan Breast Cancer Panels 1–4 (Origene Technologies, Inc). The relative expression of *FAM83A* between normal associated (N) and cancerous (C) tissue is presented. The statistical significance was determined using a Welch’s t-test, as described previously [31].

### Cell lines and culture conditions

All cell lines were grown in a humidified atmosphere containing 5% CO_2_ at 37°C. BT474, BT474 TrasR, SKBR3, SKBR3 TrasR, HCC1954 were grown in Hyclone RPMI; (with L-glutamine; Fisher Scientific SH30027FS), with 10% fetal bovine serum (Atlanta Biologicals), 50 U/ml penicillin, and 50 μg/ml streptomycin sulfate (Fisher Scientific MT30001CI). BT474 TrasR and SKBR3 TrasR cells that were generated by treating cells with 100μg/ml trastuzumab for >9 months time [38] and isogenic parental controls were a kind gift from Ron Bose (Washington University School of Medicine) [37]. HCC1954 cells were obtained from ATCC (CRL-2338). BT474 and BT474 TrasR were a kind gift from Ruth Keri (Case Western Reserve University; S1 Fig) and were grown in ATCC HybriCare medium (ATCC) and supplemented with 10% fetal bovine serum. HEK293T cells were used to produce virus, as previously described [39] and grown in DMEM (with glucose and L-glutamine; Fisher Scientific MT10013CV) supplemented with 5% fetal bovine serum. All cells were routinely tested for mycoplasma using MycoAlert Mycoplasma Detection Kit (Lonza).

### Lentiviral vectors and viral infection

Plasmids encoding shRNAs targeting FAM83A or GFP in pLKO.1 were acquired from Sigma-Aldrich (Sigma Aldrich; sh83A2 TRCN0000168628, sh83A6 TRCN0000168368) [33]. FLAG-FAM83A expression construct was created using LongAMP *TAQ* PCR kit (New England Biosystems) (forward primer GGGCCGCCACCATGGACTACAAAGAC, reverse primer GGGCGGCCGCATCGATCCTGGGCCTGCGGA). Reactions included a final *Taq* extension to create A overhangs for cloning into pCR8 entry vector using pCR8/GW/TOPO TA Cloning Kit with One Shot TOP10 *E*. *coli* (Thermo Fisher Scientific). LR clonase (Thermo Fisher Scientific) was used to recombine FLAG-FAM83A into a pLenti CMV Neo Dest vector (Addgene 17392). The EGFR expression vector was created by recombining pDONR223-EGFR (Addgene 23935) with plx304 (Addgene 25890) using LR Clonase.

Lentiviral vectors were transfected into HEK293T cells using Lipofectamine 2000 (Thermo Fisher Scientific) together with second generation packaging constructs pCMV-dR8.74 and pMD2G (gifts from D. Trono, University of Geneva, Geneva, Switzerland). Supernatants containing virus were collected at 24 and 48 hours and supplemented with 4 μg/ml polybrene (Santa Cruz) before being frozen in aliquots. Cells were subsequently infected with virus for 16 hours.

### RNA isolation and real-time PCR

Total RNA was isolated using Qiagen RNeasy miniprep kit (Qiagen). Reverse transcription was performed using iScript cDNA Synthesis Kit (BioRad). iQ SYBR Green Supermix (BioRad) was utilized for real-time PCR. Analysis was performed on triplicates of each sample using a CFX96 Real-Time System (BioRad) and C(t) for all genes was calculated after 40 cycles. FAM83A forward (CTCGGACTGGAGATTTGTCC) and reverse (GGAACTCCTCGTCAAACAGC) primers were used along with GAPDH forward (TGCACCACCAACTGCTTAGC) and reverse (GGCATGGACTGTGGTCATGAG) primers. FAM83A expression levels were normalized to GAPDH.

### Relative growth assay

BT474, BT474 TrasR, HCC1954, SKBR3, and SKBR3 TrasR cells were plated in triplicate at 30,000 cells/well, and after 7 days, cell number was determined on a Beckman Coulter counter. Trastuzumab, obtained from University Hospitals Cleveland Medical Center, was added where indicated.

### Immunoblot and immunoprecipitation

Cells were lysed in NP40 lysis buffer (150mM NaCl, 1% NP-40, 50mM Tris-Cl pH 8.0) plus Protease Inhibitor Cocktail (Sigma P8340) plus PhosSTOP (Sigma 4906845001). Cell extracts containing equal quantities of proteins, determined by the Bradford method, were separated by 7.5% acrylamide SDS-PAGE gel (Mini-PROTEAN TGX Precast Protein Gel; BioRad) and transferred to polyvinylidene difluoride membranes (Millipore) or added to an immunoprecipitation reaction. For immunoprecipitation, 4μg FLAG antibody (Sigma-Aldrich; mouse; F1804) was complexed to 40μl PBS-washed protein-G Dynabeads (Thermo Fisher Scientific 10003D) in 500μl NP40 lysis buffer plus protease and phosphatase inhibitors by rotating for 2 hours at 4°C and washed twice with PBS before cleared protein lysates were added. The antibody-bead conjugates were rotated at 4°C for 3 hours, washed eight times with RIPA lysis buffer (50mM Tris HCl pH 8, 150 mM NaCl, 1% NP-40, 0.5% sodium deoxycholate, 0.1% SDS) and resuspended in 25μl of 2X SDS BME loading dye in reparation for immunoblot analysis. Immunoblot antibodies include: FLAG (monoclonal mouse; A8592) from Sigma, and antibodies to EGFR (monoclonal rabbit; 4267), p-EGFR Y1068 (polyclonal rabbit; 2234), HER2 (monoclonal rabbit; 4290), pHER2 Y1248 (polyclonal rabbit; 2247), pAkt S473 (monoclonal rabbit; 4060), pan-Akt (mouse monoclonal; 2920), ERK (monoclonal rabbit; 4695), p-ERK (monoclonal rabbit; 4370), cleaved caspase 3 (polyclonal rabbit; 9661), GAPDH (monoclonal mouse; 97166S) from Cell Signaling Technology were used for protein detection at a dilution of 1:1000. Primary antibodies were detected with goat anti-mouse or goat anti-rabbit conjugated to horseradish peroxidase (Cell Signal Technology 7076S and 7074S), using Western Lightning Plus enhanced chemiluminescence (Perkin-Elmer). Image J software was used to quantify protein expression.

### Soft agar and organotypic cultures

For soft agar assays, 1 x 10^5^ cells were suspended in 0.6% type VII agarose (Sigma-Aldrich) and plated in triplicate onto a bottom layer of 1.2% agar in 60-mm plates. Medium was changed every 3 days for 2 weeks. To quantify colonies, each plate was scanned using an automated multipanel scanning microscope and the digital images were analyzed using MetaMorph image quantification software. Laminin rich basement membrane (lrBM) (Matrigel; BD Biosciences) cultures were made by overlaying 200μl of lrBM onto 6 well chamber dishes and allowing it to solidify for 30 minutes at 37ºC in a cell culture incubator. 5×10^5^ cells were embedded in 800μl lrBM and allowed to solidify for 1 hour. The solidified cultures were fed with 1ml complete growth medium, and re-fed with 2ml growth medium every 3 days. Brightfield images were taken at day 7 and total acini were quantified by acini counts in 5 random fields per well.

### Cell cycle and apoptosis analysis

Cells were infected with titered lentiviral shRNAs for 16 hours then refreshed with media for an additional 24 hours before being selected in 2μg/ml puromycin (Sigma P8833). Cells and media supernatants were collected and analyzed at day 4–6, as indicated. Cells treated with 50μM etoposide for 16 hours were used as a positive control. For BrdU analysis, a FITC-BrdU Flow Kit (BD Pharmingen) was used per manufacturer’s instructions and analyzed using a BD LSR II flow cytometer. For Annexin V staining, cells and media supernatant were collected for analysis using an eBiosciences Annexin V Apoptosis Detection Kit FITC (eBiosciences) per manufacturer’s instructions and analyzed using a BD LSR II flow cytometer. For Propidium Iodide staining for cell cycle analysis, cells and media supernatant were collected, washed with PBS and fixed by dropwise addition of cold 70% ethanol while vortexing, then stored at -20°C for a minimum of 1 hour. Fixed cells were washed twice with PBS, incubated in 100μl of 20μg/ml RNAse A in PBS at 37°C for 30 minutes, then chilled at 4°C for 10 minutes. Propidium iodide was added to the suspension (100μl of 100μg/ml PI) for a final concentration of 50μg/ml and the cells were covered and incubated at 4°C for 60 minutes, strained into flow tubes and analyzed using an Attune NXT flow cytometer.

## Results

### FAM83A is overexpressed in HER2+ BC

**Fig 1:**
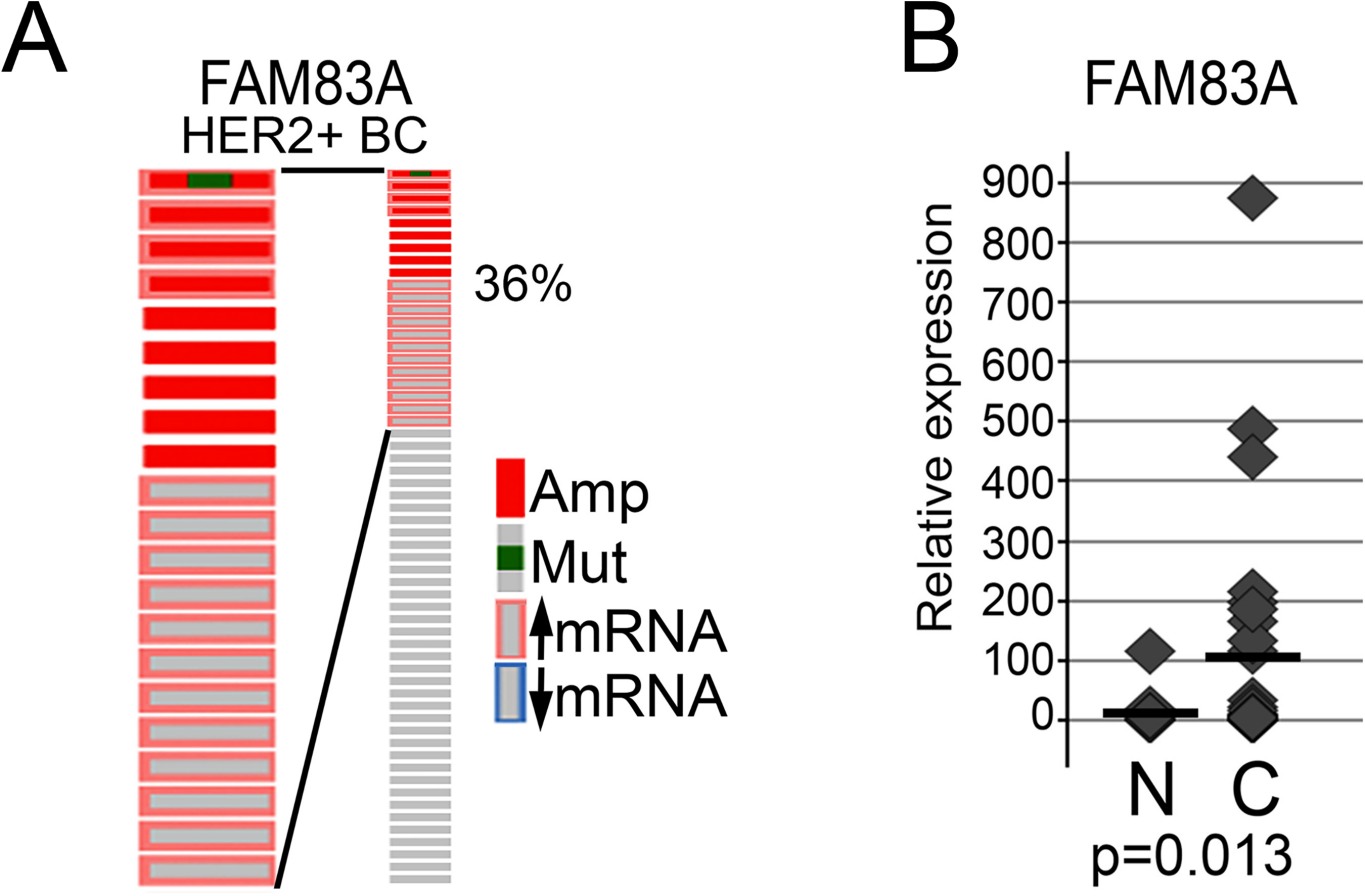
FAM83A expression is elevated in HER2+ BC. (A) cBioportal analysis of FAM83A expression in 58 HER2+ BC patients in the TCGA dataset. Amp-gene amplified, Mut-gene mutated. (B) FAM83A expression in HER2+ patient breast cancers compared to normal breast tissue using an Origene TissueScan qPCR array show FAM83A is significantly elevated in HER2+BC tumors (p = 0.013). N-normal, C-HER2+ BC.

To examine the physiological relevance of FAM83A in HER2+ BC, perturbations in FAM83A were assessed using cBioPortal to analyze TCGA data [40, 41] (Fig 1A). Of 59 HER2+ BC tumors, the FAM83A gene is amplified or overexpressed in 36% of cases. Analysis of one patient sample revealed a V142D missense mutation in the Domain of Unknown Function 1669 (DUF1669) of FAM83A, a conserved domain among FAM83 members that is required for oncogenic transformation. While we do not know whether the FAM83A-V142D mutation affects FAM83A protein function, it is clear that among HER2+BCs and across many cancer types, that recurrent FAM83A mutations are not prevalent. Rather, elevated FAM83A expression is the most frequent alteration to the FAM83A locus. Additional analysis of FAM83A expression in 45 HER2+ BC and 13 normal breast tissues using quantitative real-time PCR (qPCR) further confirmed that FAM83A is frequently overexpressed in HER2+ BC (Fig 1B). Our findings suggest that FAM83A may be a physiologically relevant oncogene in this aggressive breast cancer subtype.

### Elevated FAM83A expression correlates with trastuzumab resistance

**Fig 2:**
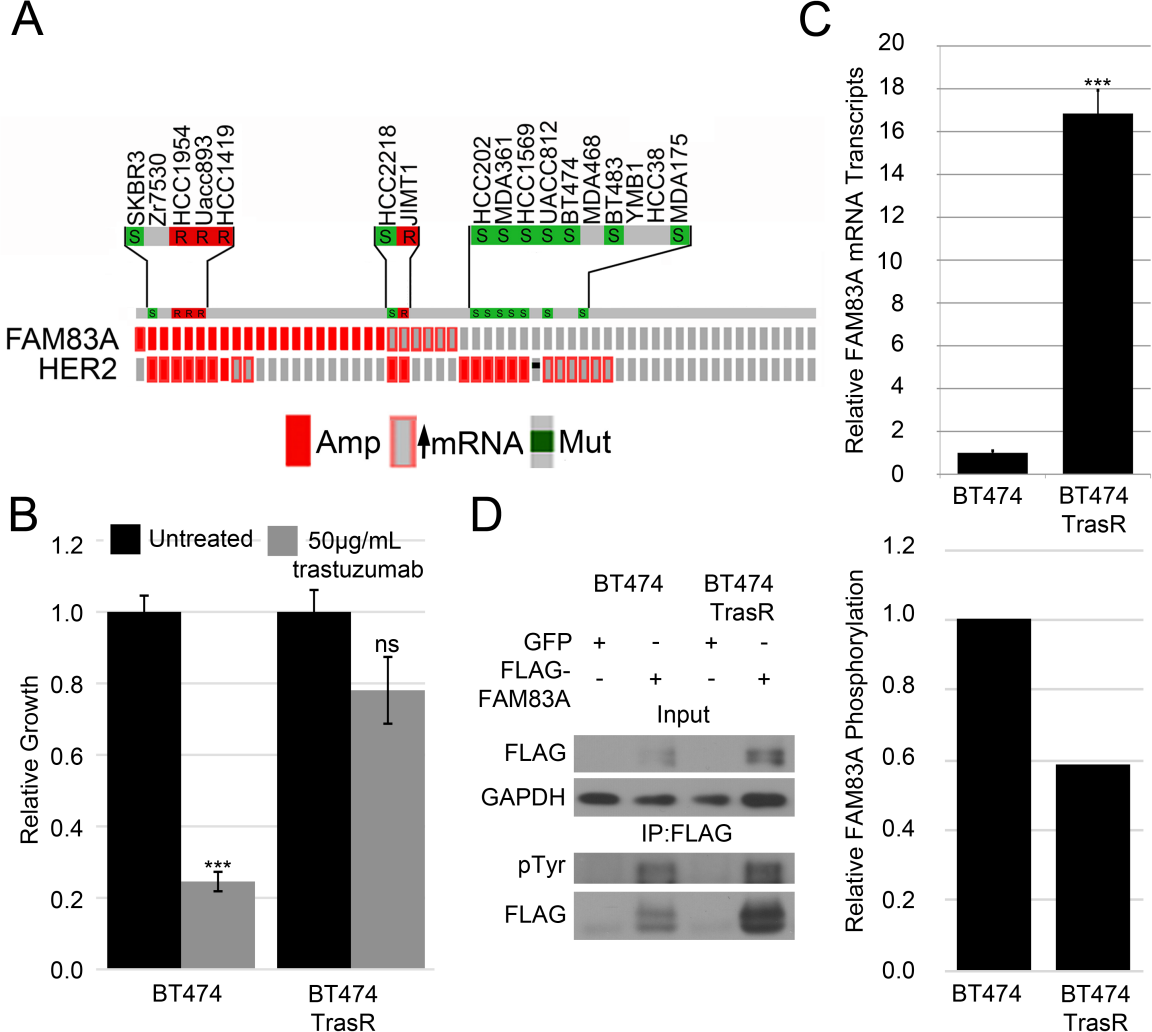
Elevated FAM83A expression correlates with trastuzumab resistance. (A) FAM83A expression in HER2+ BC cells in the Cell Line Encyclopedia dataset shows elevated FAM83A expression correlates with trastuzumab-resistant cells. S–trastuzumab-sensitive, R- trastuzumab-resistant (B) Relative growth assay of BT474 and BT474 TrasR cells in trastuzumab confirms the BT474 TrasR cells are resistant to trastuzumab. Depicted are standard deviations of 3 technical replicates, representative of 3 biological replicates. (C) Real-time PCR analysis of FAM83A in BT474 and BT474 TrasR cells indicates FAM83A mRNA expression is elevated in BT474 TrasR cells. Error bars represent the standard error of the mean. (D) Phospho-tyrosine analysis of FAM83A immunoprecipitated from BT474 or BT474 TrasR cells demonstrates decreased relative pTyr phosphorylation of FAM83A in BT474 TrasR cells. *ns–*not significant, *p<0.05, **p<0.01, ***p<0.001, Student’s t-test.

Because of its role in EGFR TKI resistance and its elevation in HER2+ BC, we sought to investigate whether FAM83A might also play a role in trastuzumab resistance. Previous work performed by Boyer *et al*. indicated FAM83A is hyper-tyrosine phosphorylated in HER2+ BC cells selected for trastuzumab resistance. From their studies, siRNA-mediated knockdown of FAM83A expression led to re-sensitization of trastuzumab-resistant cells to the drug. To determine the role of FAM83A in trastuzumab-resistance, FAM83A expression was examined in HER2+ BC cell lines using the Cell Line Encyclopedia database [42] and correlated with published data on trastuzumab sensitivity, where available [43–46] (Fig 2A). Interestingly, elevated FAM83A expression was common in trastuzumab resistant cells, including HCC1954, UACC893, MDA453, HCC1419, and JIMT1. In contrast, trastuzumab-sensitive cells such as BT474, SKBR3, HCC2218, HCC202, MDA361, HCC1569, UACC812, BT483, and MDA175 do not have elevated FAM83A expression.

To further define the role of FAM83A in trastuzumab resistance, parental, trastuzumab-sensitive BT474 cells and a trastuzumab-resistant derivative (BT474-TrasR) were obtained [37]. The BT474-TrasR cells were derived by growing parental BT474 cells in 100μg/ml of trastuzumab for >9 months and confirmed to be resistant to trastuzumab (Fig 2B). As mentioned, FAM83A was identified as being hyper-tyrosine phosphorylated in these trastuzumab-resistant cells [37]. However, it remains unclear whether this is due to increased phosphorylation of FAM83A protein or increased FAM83A expression in the BT474-TrasR cells. Using qPCR, we observed that FAM83A expression is >15-fold higher in the BT474-TrasR cells relative to parental BT474 cells (Fig 2C). Additionally, FAM83A expression is elevated in a second, alternatively derived trastuzumab-resistant BT474 population (S1 Fig). However, we observed no increase in FAM83A tyrosine phosphorylation in BT474 TrasR cells when exogenously expressed FLAG-tagged FAM83A was immunoprecipitated from parental BT474 or BT474-TrasR cells and probed with a phospho-tyrosine antibody (Fig 2D). When normalized to total immunopreciptated FLAG-FAM83A, we observed a 41.5% decrease in relative pY-FAM83A in the BT474 TrasR cells. This indicates the increased FAM83A phosphorylation level noted by Boyer *et al*. was due to an overall increase in FAM83A protein, rather than an increased ability of a tyrosine kinase to phosphorylate FAM83A protein. Taken together, our findings suggest that elevated FAM83A expression is selected for in trastuzumab resistant HER2+ BC, including cells originating from trastuzumab-resistant HER2+ BC patients and cells selected for resistance by long term growth in trastuzumab.

### FAM83A is not sufficient to confer trastuzumab resistance

**Fig 3:**
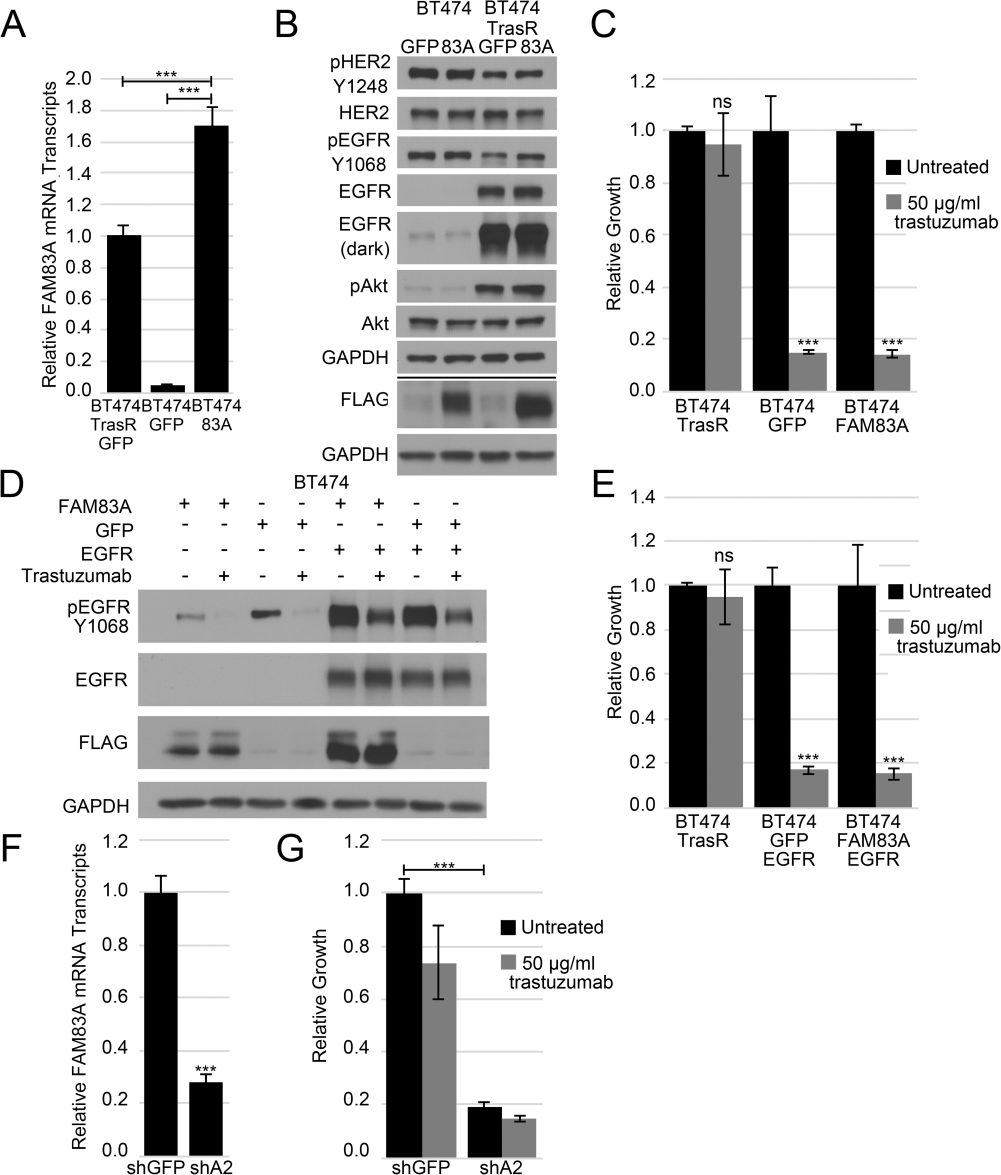
FAM83A in the trastuzumab resistant phenotype. (A) Real-time PCR analysis of FAM83A mRNA expression in BT474 cells expressing exogenous FAM83A or control GFP and BT474 TrasR cells indicates exogenous BT474 FLAG-FAM83A expression is increased over endogenous BT474 TrasR cells. Error bars represent standard error of the mean. (B) Immunoblot analysis of pHER2 Y1248, HER2, pEGFR Y1068, EGFR, and FLAG in BT474 FLAG-FAM83A and control GFP and BT474 TrasR FLAG-FAM83A and GFP cells. (C) Relative growth assay of BT474 FLAG-FAM83A and GFP and BT474 TrasR cells in 10μg/ml trastuzumab reveals no change in trastuzumab sensitivity upon exogenous FAM83A expression. Error bars represent standard deviations of 3 technical replicates. (D) BT474 FLAG-FAM83A and GFP cells were transduced with exogenous EGFR. Immunoblot shows p-EGFR Y1068, EGFR, and FLAG expression in the presence or absence of trastuzumab and indicates activation of the exogenous EGFR. (E) Relative growth assay of BT474 FLAG-FAM83A/EGFR and BT474 GFP/EGFR and BT474 TrasR cells shows neither exogenous expression of EGFR alone nor in conjunction with FAM83A is sufficient to confer trastuzumab resistance. Error bars represent the standard deviation of triplicate technical replicates. (F) BT474 TrasR cells were transduced with an shRNA targeting GFP or FAM83A. Real-time PCR analysis of FAM83A mRNA expression illustrates reduced FAM83A expression in shFAM83A cells. Error bars represent standard error of the mean. (G) Relative growth assay of BT474 TrasR shFAM83A or shGFP cells untreated or treated with trastuzumab demonstrates a stark growth inhibition in shFAM83A cells, regardless of trastuzumab treatment. Error bars represent standard deviations of 3 technical replicates. *ns–*not significant, *p<0.05, **p<0.01, ***p<0.001, Student’s t-test.

Elevated FAM83A expression can promote resistance to EGFR TKIs; therefore, we hypothesized that the elevated FAM83A expression may also promote resistance to trastuzumab. To test this hypothesis, the parental BT474 cells expressing exogenous FLAG-tagged FAM83A or GFP (as a control) shown in Fig 2D were assessed for trastuzumab sensitivity. qPCR confirmed that FAM83A was elevated 33-fold in BT474-FAM83A cells relative to GFP-expressing controls (Fig 3A). By comparison, the BT474-FAM83A cells expressed 1.7-fold more FAM83A mRNA than is endogenously expressed in the BT474-TrasR cells. Of note, no change was observed in HER2/pHER2 or EGFR/pEGFR expression levels in BT474-FAM83A cells (Fig 3B). To assess whether elevated FAM83A expression confers trastuzumab resistance, growth assays were performed in the presence or absence of trastuzumab. Surprisingly, the BT474-FAM83A cells exhibited equal sensitivity to trastuzumab as the BT474-GFP control cells (Fig 3C). Thus, FAM83A alone is not sufficient to confer trastuzumab resistance.

BT474-TrasR cells also express elevated levels of EGFR and pAkt (Fig 3B). Increased EGFR activity has been linked to trastuzumab resistance and FAM83A was previously described to interact with the EGFR pathway [32]. Yet, despite expressing high levels of FAM83A mRNA and protein, no change was observed in HER2/pHER2 or pEGFR/EGFR expression levels compared to controls (Fig 3B). Therefore, we hypothesized that elevated expression of both EGFR and FAM83A may be necessary to drive trastuzumab resistance. To test this hypothesis, EGFR was expressed in parental, trastuzumab-sensitive BT474 cells in the presence or absence of FLAG-FAM83A (Fig 3D). Immunoblot analysis also confirmed that the high levels of EGFR were tyrosine-phosphorylated, indicating the exogenously expressed EGFR was activated. However, none of the EGFR- or FAM83A-expessing derivatives displayed a decrease in trastuzumab-sensitivity (Fig 3E). Thus, despite robust overexpression of both FAM83A and EGFR to levels even greater than endogenous levels observed in BT474-TrasR cells, the trastuzumab resistance could not be recapitulated. These results indicate that while FAM83A is overexpressed in TrasR cells, overexpression of FAM83A alone or with EGFR in trastuzumab-sensitive cells is not sufficient to confer trastuzumab resistance. Of note, despite the increased levels of endogenous EGFR protein in BT474 TrasR cells, they are only modestly resistant to the EGFR TKI erlotinib when compared to parental BT474 (S2 Fig, single data values). Although FAM83A is not sufficient for resistance, the high level of FAM83A expression in BT474 TrasR cells remains intriguing.

To determine if FAM83A is necessary for trastuzumab resistance, lentiviruses encoding FAM83A- or GFP-targeting shRNAs were used to stably infect BT474-TrasR cells. Quantitative RT-PCR analysis confirmed a 75% knockdown of FAM83A mRNA expression when compared to the shGFP control (Fig 3F). Upon FAM83A knockdown, a remarkable 80% reduction in cell number was observed, irrespective of whether or not the cells were treated with trastuzumab (Fig 3G). The substantial reduction in cell number following FAM83A ablation renders trastuzumab sensitivity assessment difficult, but from our results we observe no significant difference in trastuzumab sensitivity between the shFAM83A2-expressing and control BT474 TrasR cells (Fig 3G). Knockdown of FAM83A also failed to increase sensitivity to EGFR therapies in these cells including erlotinib (S3A Fig) and lapatinib (S3B Fig), despite previous work showing FAM83A knockdown re-sensitizes TNBC cell lines to these therapies.

### FAM83A is required for HER2+ BC growth

**Fig 4:**
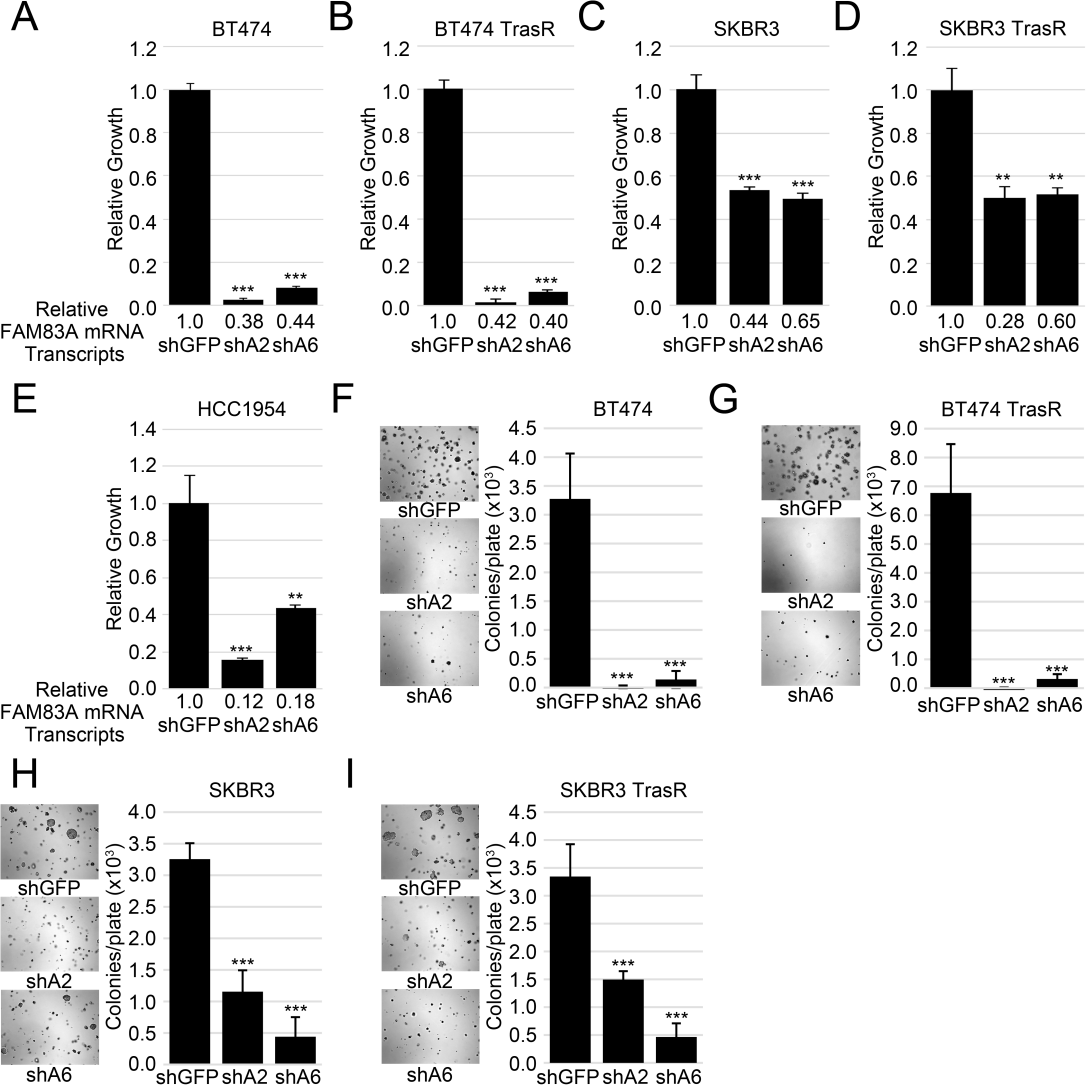
Knock-down of FAM83A expression inhibits HER2+ BC cell growth. BT474 (A), BT474 TrasR (B), SKBR3 (C), SKBR3 TrasR (D), and HCC1954 (E) cells were transduced with two different shRNAs targeting FAM83A and a control shRNA targeting GFP. FAM83A mRNA expression levels are indicated below each graph. All cell lines were plated in triplicate. Error bars represent the standard deviation. BT474 (F), BT474 TrasR (G), SKBR3 (H), and SKBR3 TrasR (I) expressing shGFP, shA2, or shA6 were assessed for anchorage independent growth in soft agar colonies. Images were taken and colonies were counted using MetaMorph software. Original magnification 5X. HCC1954 cells did not grow in soft agar. *ns–*not significant, *p<0.05, **p<0.01, ***p<0.001, Student’s t-test.

Given the strong suppression of growth following FAM83A ablation, we hypothesized that FAM83A could have a more global impact on the growth of HER2+ BC cells. Analyses were expanded to include a panel of HER2+ cell lines including BT474 and SKBR3 cells, both trastuzumab sensitive and resistant derivatives, as well as HCC1954 cells, which are naturally resistant to trastuzumab. Again, FAM83A expression was knocked-down using lentiviral delivery of shRNAs (Fig 4A–4E). Knockdown efficiency ranged from 35%-90% reduction in FAM83A mRNA, and the knockdown of FAM83A remained stable up to 15 days post lentiviral infection (S4 Fig). Unfortunately, all currently available commercial antibodies were unable to detect even exogenously expressed FLAG-FAM83A protein. However, we did verify the efficacy of shA6, which strongly inhibited the expression of exogenous FLAG-FAM83A in both BT474 and BT474 TrasR cell lines (S5 Fig). shA2 targets the 3’ UTR and thus does not target the exogenous FLAG-FAM83A. Although BT474 TrasR cells have significantly elevated mRNA expression of FAM83A compared to the other cell lines (S6A Fig), all HER2+ BC lines have elevated FAM83A mRNA expression compared to normal diploid human fibroblasts (S6B Fig). In all cell lines tested, knockdown of FAM83A expression inhibited cell growth, from 47% in SKBR3 cells to 92% in BT474 and BT474-TrasR cells (Fig 4A–4E). Moreover, knockdown of FAM83A expression inhibited anchorage-independent growth (AIG) in soft agar (Fig 4F–4I) and growth in laminin rich basement membrane (lrBM) in all cell lines tested (S7 Fig). HCC1954 cells did not grow anchorage independently, and could not be assessed for AIG. Thus, FAM83A knockdown inhibits growth across a range of FAM83A expression levels, regardless of trastuzumab sensitivity. This finding implicates FAM83A as a potential drug target in a wider spectrum of HER2+ BC patients, including those who have progressed following trastuzumab treatment.

### Loss of FAM83A results in cell death through apoptosis

**Fig 5:**
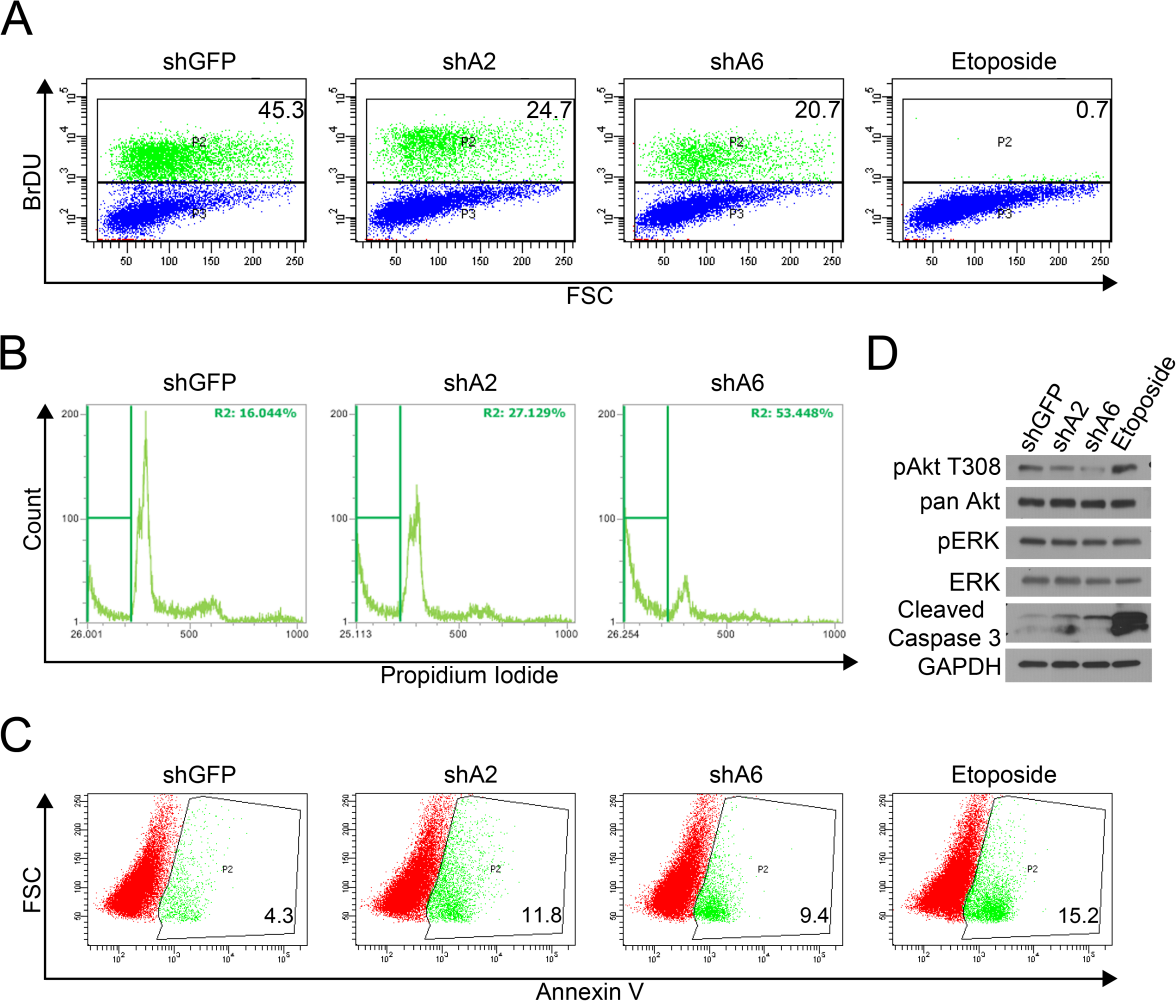
Loss of FAM83A expression induces apoptosis and inhibits PI3K/Akt signaling. HCC1954 cells infected with shGFP, shA2, or shA6 were assessed for induction of apoptosis using etoposide treated HCC1954 shGFP cells as a positive control. (A) FAM83A knock-down resulted in reduced BrdU incorporation. (B) Cells were fixed and stained with propidium iodide for cell cycle analysis. FAM83A-knockdown cells have increased sub-G_1_ peaks, indicative of cell death. (C) Live cells were stained with an antibody detecting the early apoptosis marker Annexin V. Knockdown of FAM83A resulted in elevated expression of Annexin V. (D) Immunoblot analysis of the apoptotic marker cleaved caspase 3 and cell signaling proteins shows FAM83A knockdown induces apoptosis while decreasing PI3K/Akt signaling.

To further investigate the biological consequence of FAM83A knockdown, we assessed proliferation and apoptosis in HCC1954 cells. Bromodeoxyuridine (BrdU) incorporation was assessed 6 days post-lentiviral infection, as cell growth was only modestly decreased at earlier timepoints (S8 Fig). BrdU positivity following FAM83A knockdown decreased by 45.5% and 54.5% in shA2 and shA6 cells, respectively, indicating that these cells have undergone a reduction in proliferation (Fig 5A). In addition, cells stained with propidium iodide (PI) to assess DNA content have a reduced G2 peak and an increased sub-G1 population, indicative of cells undergoing apoptosis following FAM83A knock-down (Fig 5B). Furthermore, cells stained with Annexin V, an early apoptosis marker, confirmed that FAM83A knock-down increased Annexin V staining from 4.3% in control cells to 11.8% in shA2 expressing cells and 9.4% in shA6-expressing cells (Fig 5C). Consistent with the increased sub-G1 PI staining and increased Annexin V staining, knockdown of FAM83A also induced caspase 3 cleavage, as observed by immunoblot (Fig 5D). Finally, we also observed a decrease in Akt phosphorylation, suggesting that FAM83A knockdown impacts PI3K/AKT proliferative and survival signaling similarly in HER2+ and TNBC cells [32]. The decrease in p-Akt upon FAM8A knockdown was also observed in BT474 TrasR cells (S9 Fig). Interestingly, phosphorylation of ERK was not impaired by FAM83A knockdown in HCC1954 cells, implicating that FAM83A may signal primarily through the PI3K/Akt pathways in HER2+ BC as opposed to signaling through both the MAPK and PI3K/Akt pathways in TNBCs [32, 33].

## Discussion

Trastuzumab has improved survival rates for patients with HER2+ BC, yet *de novo* and acquired resistance remain major unmet clinical needs for many patients [16, 18, 19, 28]. Mechanisms of trastuzumab resistance continue to emerge and development of new therapies to combat trastuzumab resistance remains slow [28]. FAM83A is a member of a novel oncogene family recently discovered by our lab, together with the laboratory of Mina Bissell, based on its ability to drive HMEC transformation and EGFR TKI resistance [31–33]. Here we show that FAM83A expression is upregulated in HER2+ BCs, including those that are resistant to the HER2-targeted therapy trastuzumab. The increased FAM83A expression is supported by a proteomics study that identified FAM83A as one of the most highly tyrosine-phosphorylated proteins in trastuzumab-resistant cells [37]. Moreover, analysis of data from trastuzumab-resistant and sensitive cell lines available in the Cell Line Encyclopedia [42] identified a correlation between cells harboring amplified or overexpressed FAM83A and trastuzumab resistance. It is important to note, however, that our analysis of FAM83A in the current study has relied solely on mRNA expression, as we have been unable to find an antibody capable of recognizing FAM83A (even after exogenous overexpression). Thus, while a recent large-scale genomics/proteomics analysis found an 86.4% correlation between mRNA and the protein it encodes, development of FAM83A antibodies will be needed to confirm that FAM83A mRNA and protein expression correlate as expected [47].

Given the observation that elevated FAM83A expression conferred resistance to EGFR TKIs in TNBC [32], we initially hypothesized that FAM83A may also confer resistance to targeted therapies for other ErbB members, such as trastuzumab. Yet, exogenous FAM83A expression was unable to confer trastuzumab resistance to sensitive BT474 cells, even when combined with exogenous EGFR expression. There are several possible explanations for this finding. One is the length of time necessary to develop resistance. Parental BT474 cells were selected in trastuzumab for greater than 9 months in order to generate resistant cells. During this time, the selection for increased proliferative signaling can yield cells that have also acquired “passenger” mutations or alterations in gene expression that do not contribute to the resistant phenotype. Indeed, FAM83A is located next to c-MYC at the chromosomal locus 8q24, which is often amplified in cancers. In addition, the stress of therapy can induce global changes in the transcriptional profile of the emerging, resistant cells. For example, CD44^Hi^/CD24^LO^ breast cancer stem cells (CSCs) are intrinsically resistant to trastuzumab [26]. Beyond the resistance to trastuzumab, CD44^Hi^/CD24^LO^ cells have a mesenchymal phenotype that coincides with a global EMT program [48]. It is possible that FAM83A is part of a larger CSC or mesenchymal signaling program, a concept we are currently examining. Beyond global transcriptional changes, there are a number of trastuzumab resistance mechanisms described, which currently makes the development of therapies to combat resistance difficult. In our study, we were surprised that the ablation of FAM83A suppressed the growth of all HER2+ BCs tested regardless of trastuzumab sensitivity or resistance, making it a promising target for HER2+ BCs harboring elevated levels of FAM83A.

Interestingly, FAM83A-E can drive HMEC transformation and both FAM83A and FAM83B have been reported to enhance the MAPK and PI3K/Akt pathways [31–33, 49]. As described using TNBC cells, knockdown of FAM83A, FAM83B, or FAM83D suppresses growth and tumorigenicity [31–33]. Because FAM83A is elevated in both HER2+ BC and TNBC (which generally harbor elevated EGFR signaling), we speculated that FAM83A plays a role in ErbB receptor signaling. FAM83A may regulate a unique aspect of HER2+ BC biology, as it is the only FAM83 member increased in HER2+ BC. FAM83A is overexpressed in 36% of HER2+ BCs, but is rarely mutated, suggesting that HER2+ BCs select for elevated FAM83A expression. Here, we show that shRNA-mediated knockdown of FAM83A results in decreased PI3K/Akt signaling, inhibited two-dimensional and three-dimensional cell growth, and induced apoptosis in a panel of HER2+ BC cell lines, regardless of trastuzumab sensitivity. Unexpectedly, shRNA-mediated FAM83A knockdown in HER2+ BC appears to primarily inhibit signaling through the PI3K/Akt pathway compared to TNBCs, where FAM83A knockdown decreases both PI3K/Akt and MAPK signaling [32, 33]. HER2+ BCs tend to rely heavily on PI3K/Akt signaling, as HER2 preferentially pairs with ErbB3, which has 6 docking sites for the p85 regulatory subunit of PI3K [2, 50]. Thus, FAM83A’s signaling may shift towards this pathway in HER2+ BCs.

While the finding that FAM83A knockdown suppresses PI3K/Akt signaling in HER2+ BCs is novel, this appears to be a redundant function of FAM83 members in other cancer types [32, 49]. The most likely explanation for the redundancy observed among the FAM83 members is that they engage similar signaling through a highly-conserved N-terminal Domain of Unknown Function (DUF1669). However, FAM83 members have vastly different C-terminal regions [33]. As the smallest FAM83 member, FAM83A lacks a large unique C-terminal region. Identifying new roles for FAM83A could hint at common, redundant FAM83 member functions requiring the DUF1669. Studies of FAM83A in TNBC identified interactions between FAM83A with PI3K subunits and c-RAF and suggested that FAM83A engages both the PI3K/AKT and MAPK pathways in some cancer types [32, 33]. Along with MAPK and PI3K signaling, FAM83A may have additional, yet undefined functions in HER2+ BC cell signaling that are important for growth. Future studies will need to further analyze the role of FAM83A in cell signaling to determine how it can be targeted to potentially improve patient survival.

Previous studies also indicated phosphorylation of FAM83A is important for its signaling function [32, 37]. Tyrosine phosphorylation plays a vital role in intracellular signal transduction, often by initiating a structural change in the phosphorylated protein that facilitates the recruitment of additional signaling partners. In cancer, aberrant phosphorylation of signaling effectors, typically by receptor tyrosine kinases, drives unchecked proliferation and survival. We propose that the phosphorylation of FAM83A is an integral part of its signaling function, allowing or strengthening the recruitment of effector proteins to receptors. The kinases that phosphorylate FAM83A remain unknown. However, the Bissell group showed that FAM83A tyrosine phosphorylation increases immediately following EGF stimulation, implying that EGFR may directly phosphorylate FAM83A [32]. In addition, the Bose group found that FAM83A was hyper-tyrosine phosphorylated in HER2+ BC [37]. It will be of interest to determine whether FAM83A is phosphorylated by ErbB receptors and by downstream signaling complexes and to define how phosphorylation nucleates signaling complexes (such as those observed in the MAPK and PI3K/AKT pathways).

Identifying how FAM83A phosphorylation may regulate signaling interactions is key to future therapies aimed at targeting its function. Identifying how phospho-FAM83A is regulated presents two unique ways to potentially target FAM83A. First, identifying the kinase(s) that phosphorylate FAM83A would potentially allow the targeting of FAM83A-mediated signaling complex formation. Large phosphoproteomics studies implicated several kinases, including PDK and AKT, in phosphorylation of FAM83A [51]. Second, the adaptor function of FAM83A could be prevented by blocking key protein-protein interactions. Whereas targeting adaptor proteins has been a controversial subject in the past, more recent studies have renewed interest in targeting these protein interactions. For example, IQ motif-containing GTPase activating protein (IQGAP1) is a known MAPK adaptor [52, 53]. Tissue-penetrating peptides that block IQGAP1 and ERK interactions decreased MAPK-driven tumor growth [54]. Previous studies of the closely related family member FAM83B found that mutating lysine 230 in the DUF1669 diminished its binding to EGFR and prevented effector signaling and activation [33]. This residue is conserved in FAM83A, but further work is needed to determine if this residue is also required for FAM83A signaling adaptor interactions. Going forward, it will be of interest to identify all key signaling interactions engaged by FAM83A in HER2+ BC and determine how such interactions can be disrupted.

While this study and others provide clues as to the function of FAM83A, there are still many additional studies needed to determine if this novel oncogene will be an ideal therapeutic target for HER2+ BC patients.

## Supporting information

S1 Fig FAM83A mRNAexpression is elevated in an alternatively derived trastuzumab-resistant BT474 cell line. (TIF)

S2 Fig BT474 TrasR cells are not resistant to erlotinib. (TIF)

S3 Fig FAM83A knock-down does not increase sensitivity to erlotinib or lapatinib. (TIF)

S4 Fig FAM83A mRNA expression is suppressed at least 15 days post lentiviral shRNA infection. (TIF)

S5 Fig shRNA-mediated knockdown of exogenous FLAG-FAM83A. (TIF)

S6 Fig BT474 TrasR FAM83A mRNA expression is elevated compared to a HER2+ BC cell panel and normal fibroblasts. (TIF)

S7 Fig FAM83A expression is required for HER2+ BC cell growth in organotypic culture. (TIF)

S8 Fig BrdU staining of cells 4 days post lentiviral shRNA infection. (TIF)

S9 Fig p-Akt is reduced in BT474 TrasR FAM83A knock-down cells. (TIF)

S10 Fig Raw scans of Fig 2D immunoblot. (TIF)

S11 Fig Raw scans of Fig 3B immunoblot. (TIF)

S12 Fig Raw scans of Fig 3D immunoblot. (TIF)

S13 Fig Raw scans of Fig 5C immunoblot. (TIF)
