# Supplementary figures and images for: HER2-positive breast cancer cells expressing elevated FAM83A are sensitive to FAM83A loss
Source: PLoS One. 2017 May 2;12(5):e0176778. doi: 10.1371/journal.pone.0176778 (PMC5413028; doi:10.1371/journal.pone.0176778)

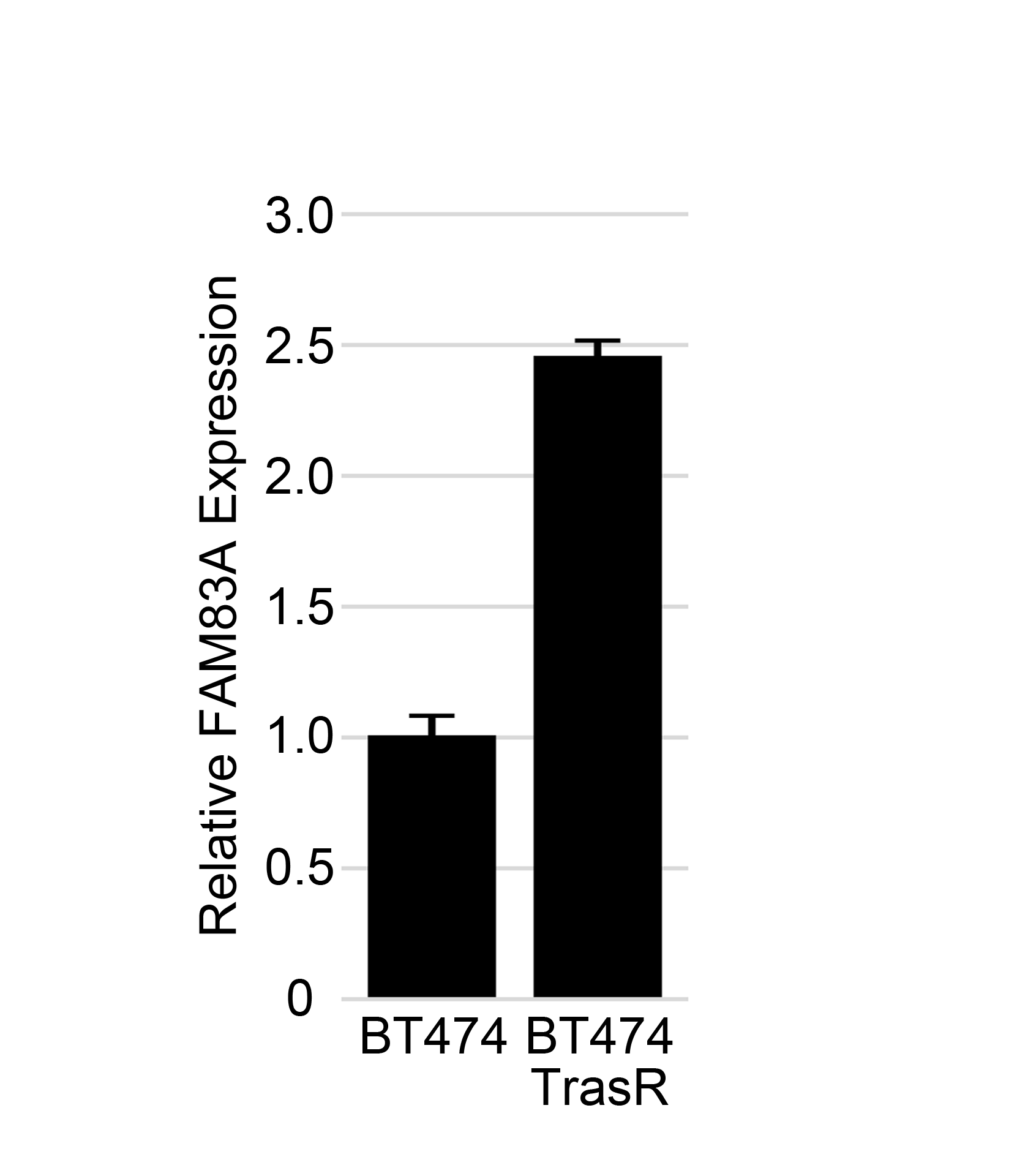

Supplement: S1 Fig — (TIF) [file pone.0176778.s001.tif]

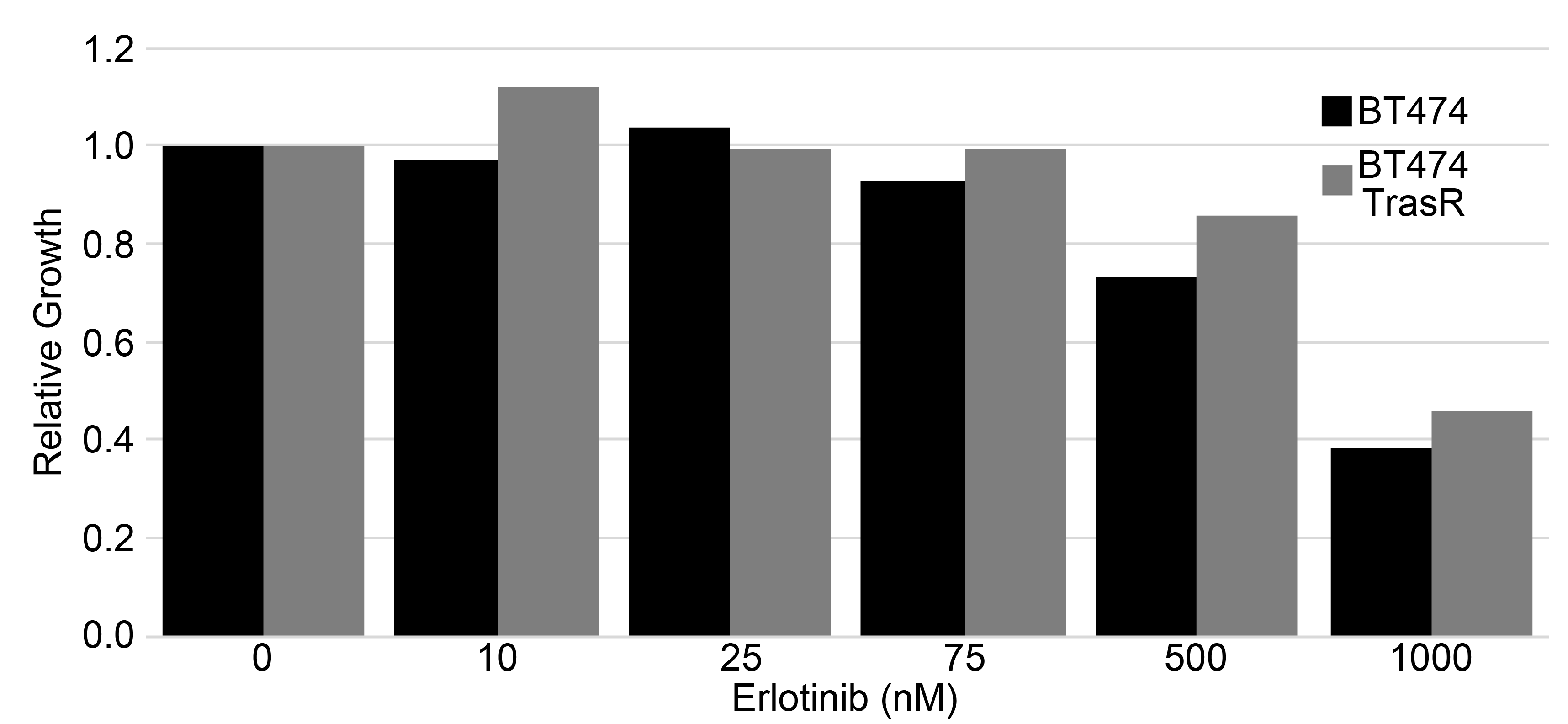

Supplement: S2 Fig — (TIF) [file pone.0176778.s002.tif]

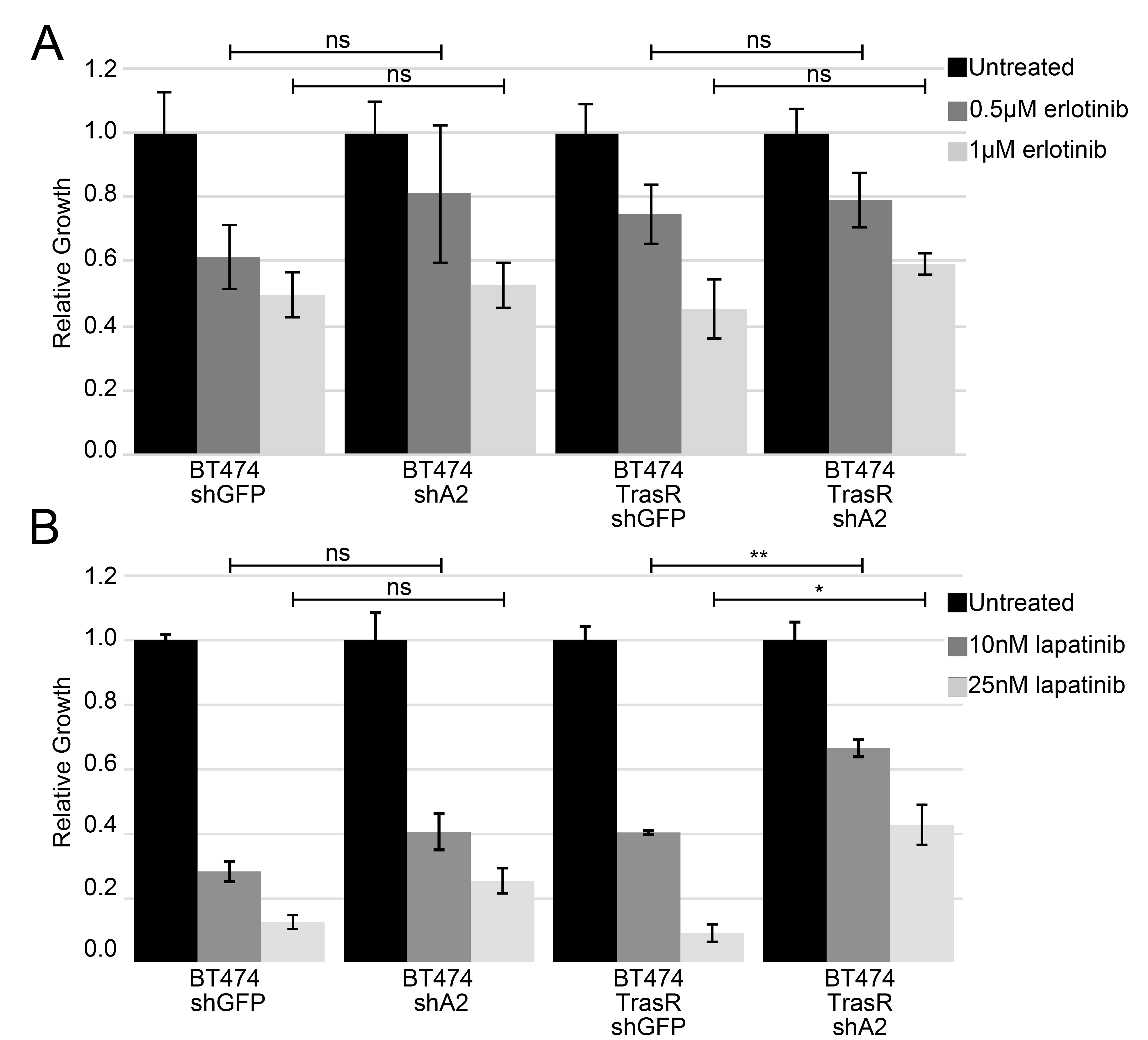

Supplement: S3 Fig — (TIF) [file pone.0176778.s003.tif]

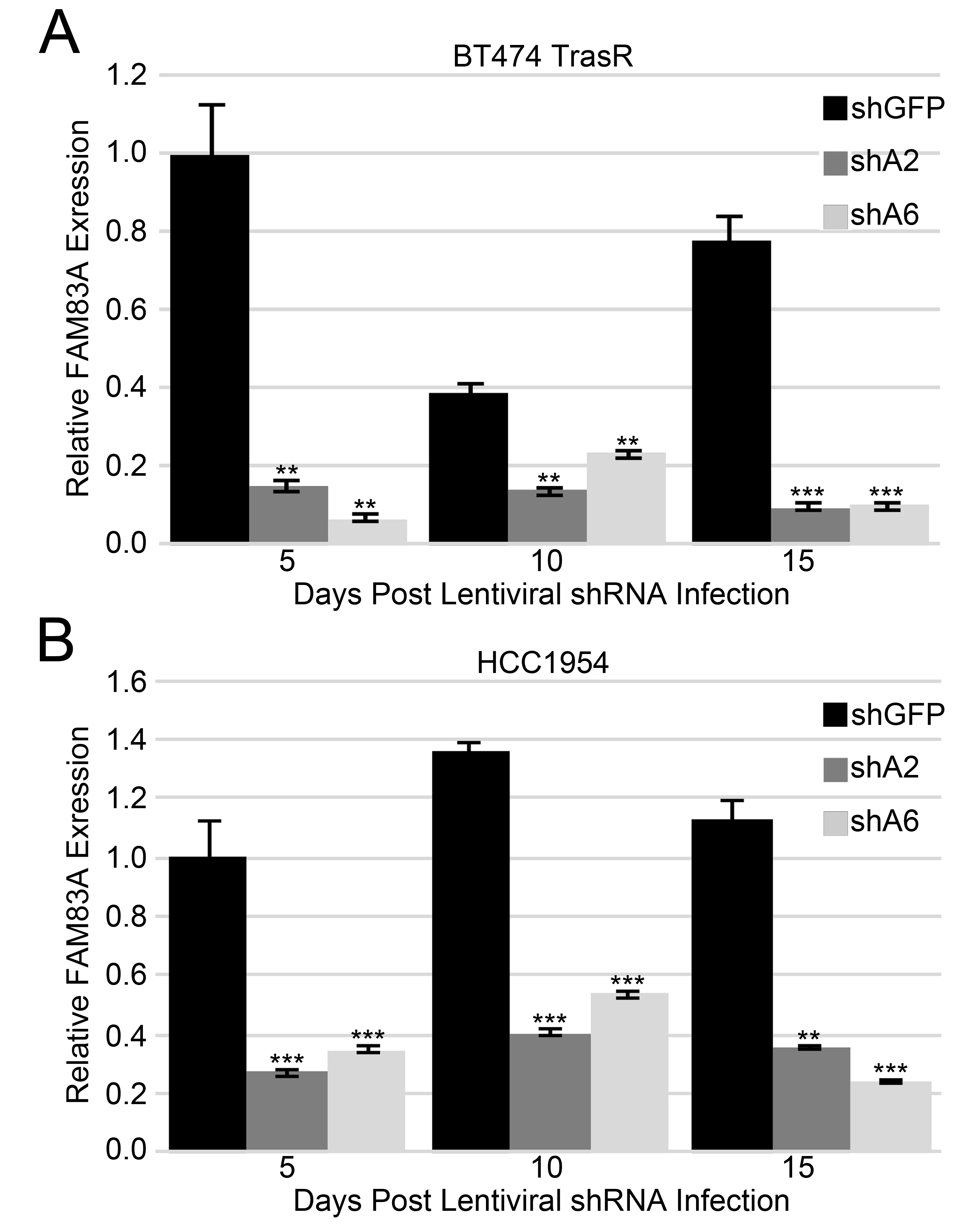

Supplement: S4 Fig — (TIF) [file pone.0176778.s004.tif]

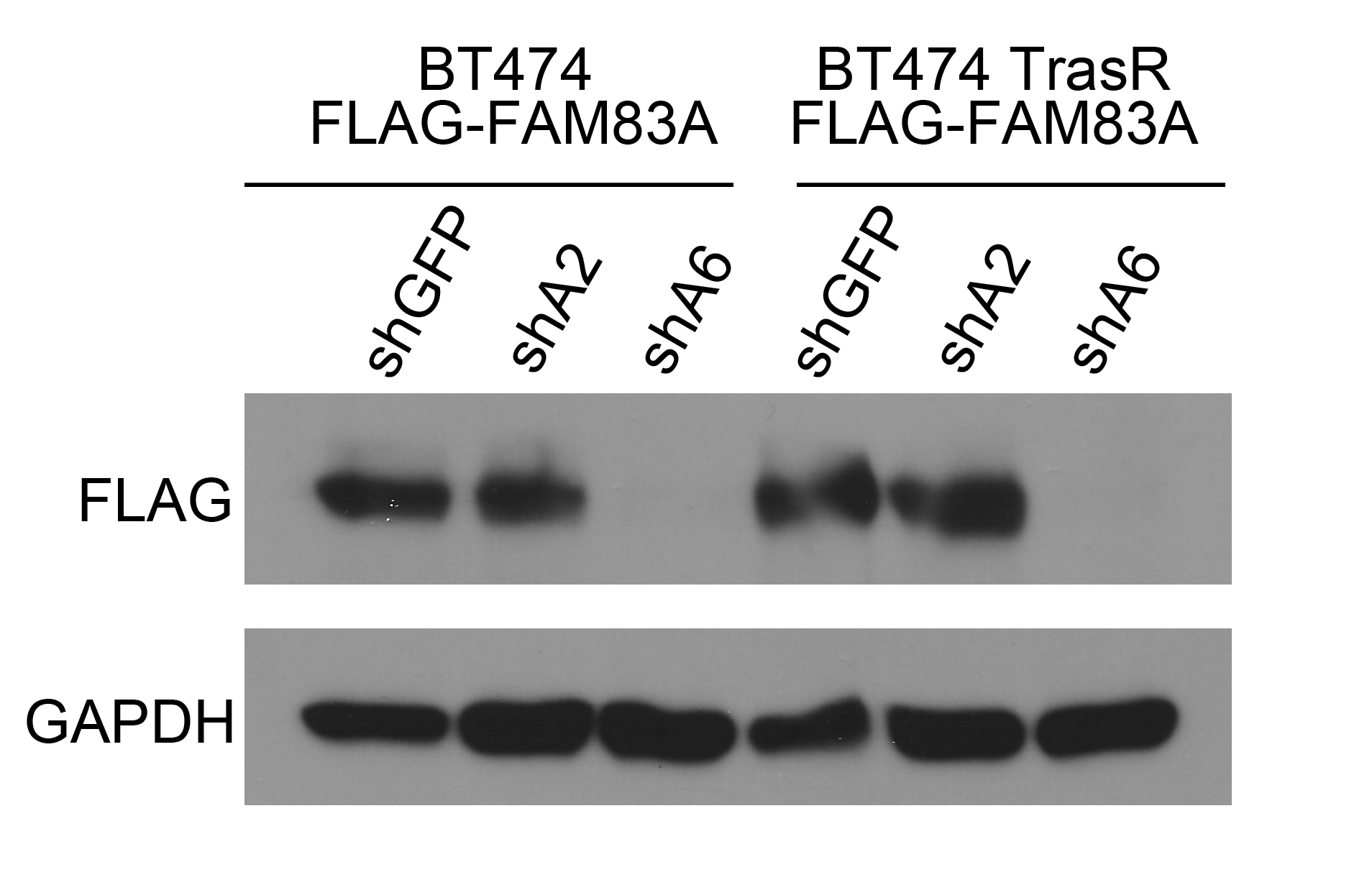

Supplement: S5 Fig — (TIF) [file pone.0176778.s005.tif]

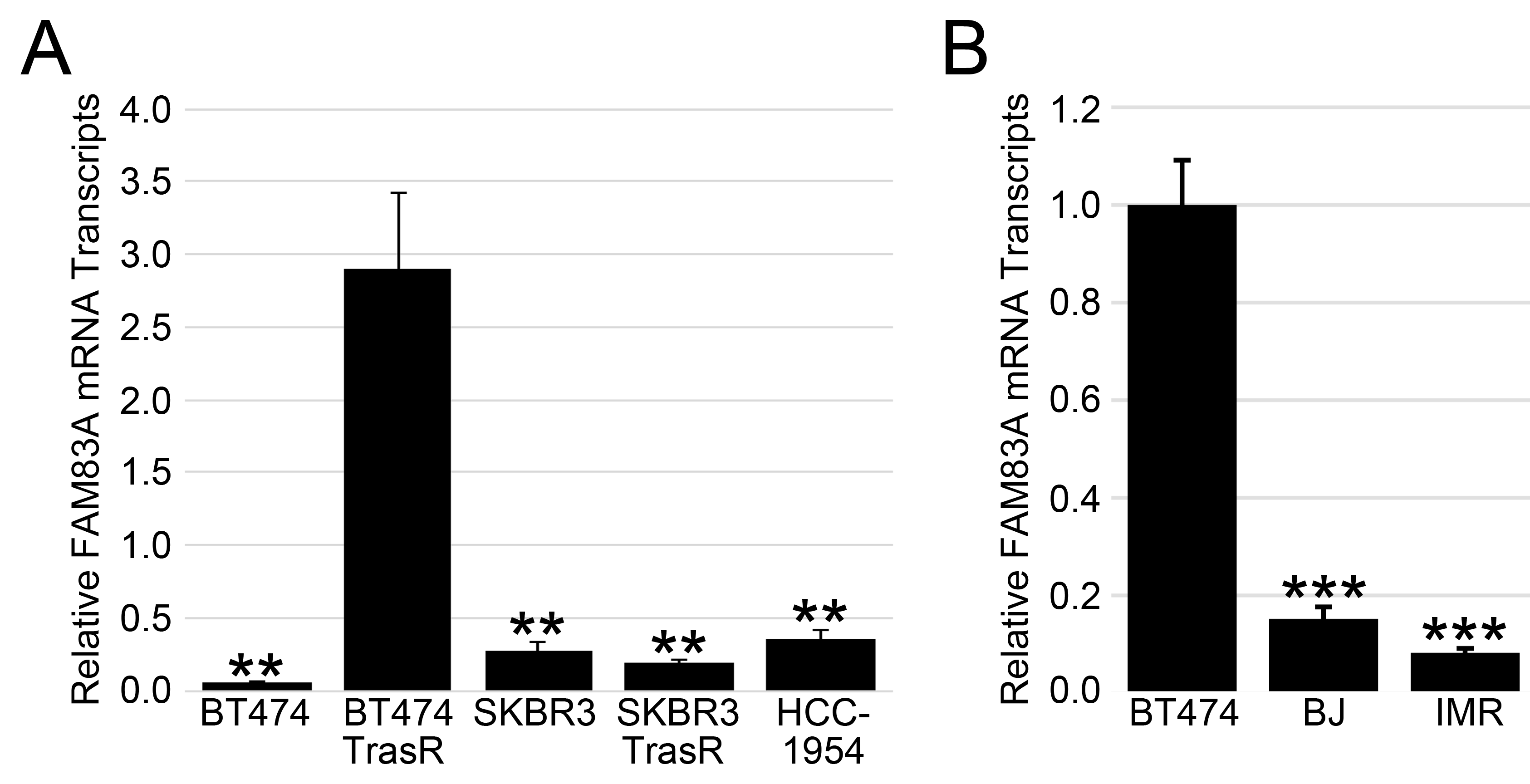

Supplement: S6 Fig — (TIF) [file pone.0176778.s006.tif]

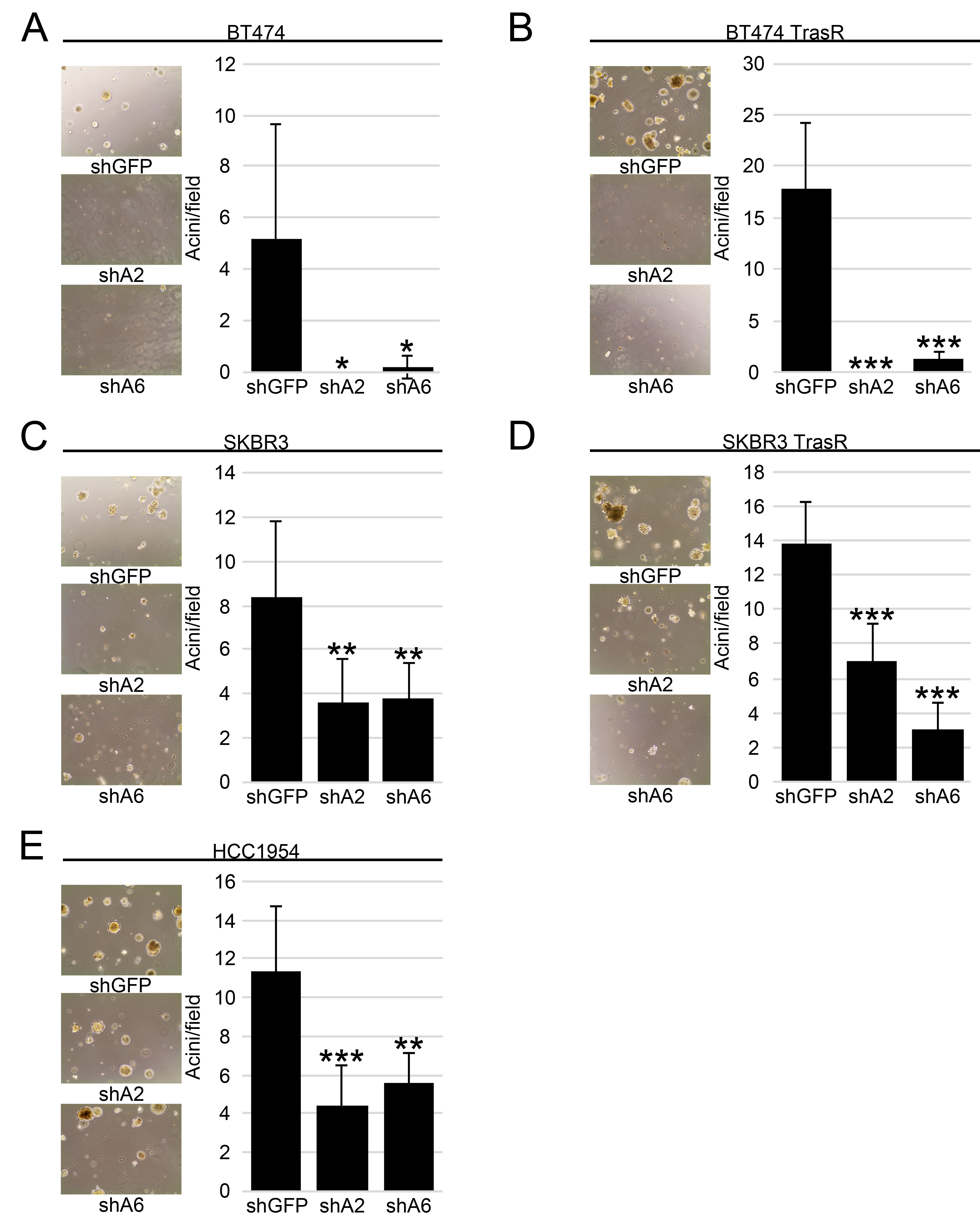

Supplement: S7 Fig — (TIF) [file pone.0176778.s007.tif]

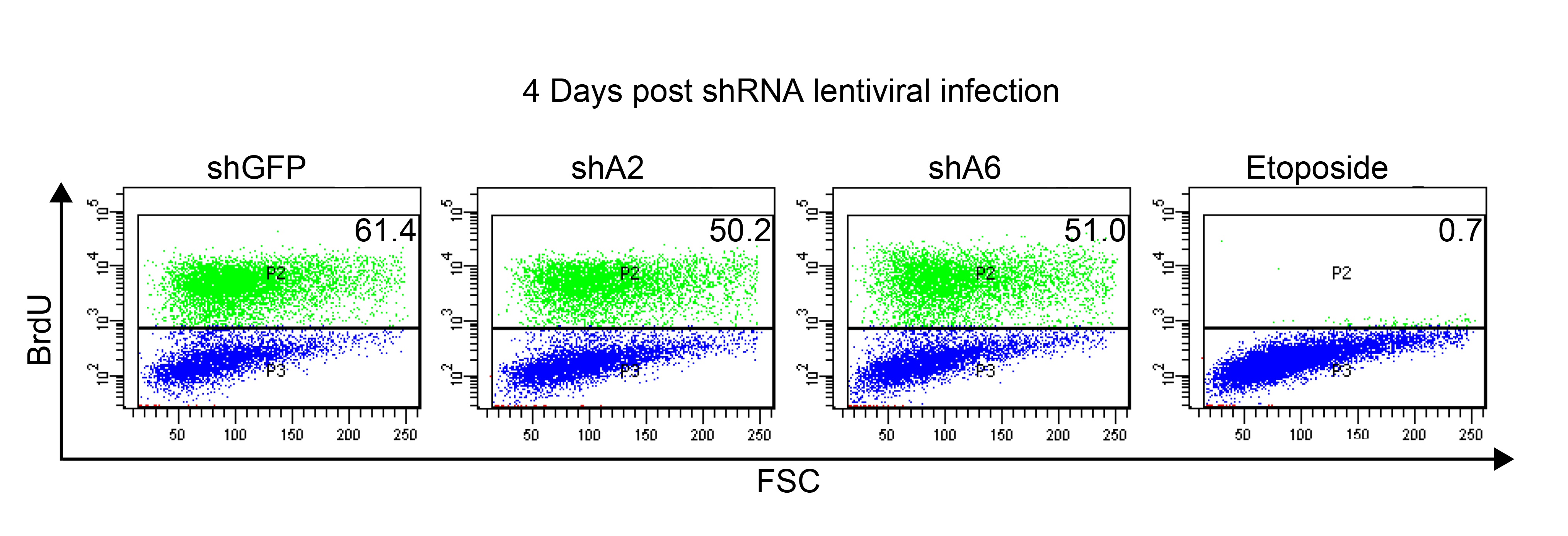

Supplement: S8 Fig — (TIF) [file pone.0176778.s008.tif]

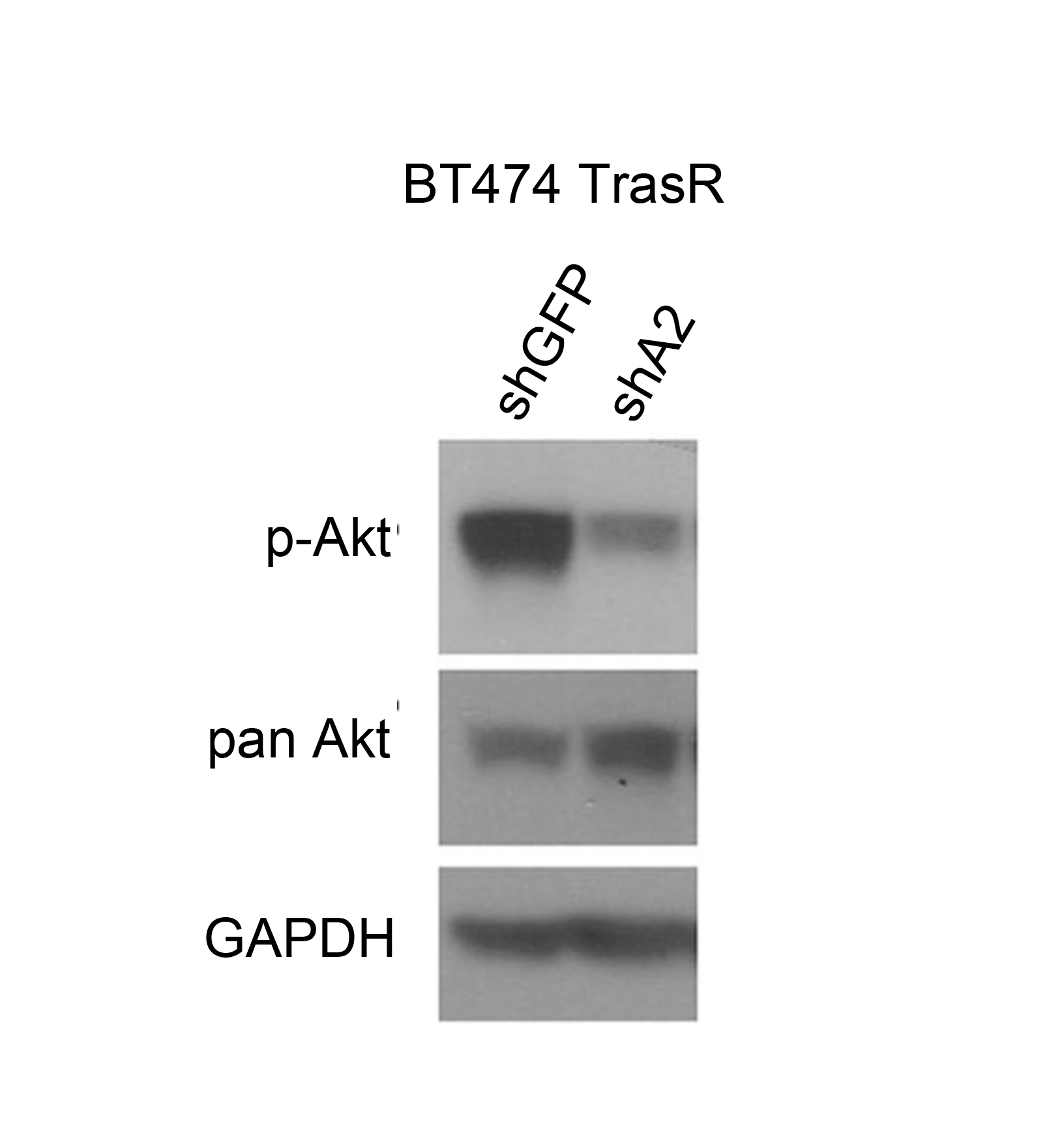

Supplement: S9 Fig — (TIF) [file pone.0176778.s009.tif]

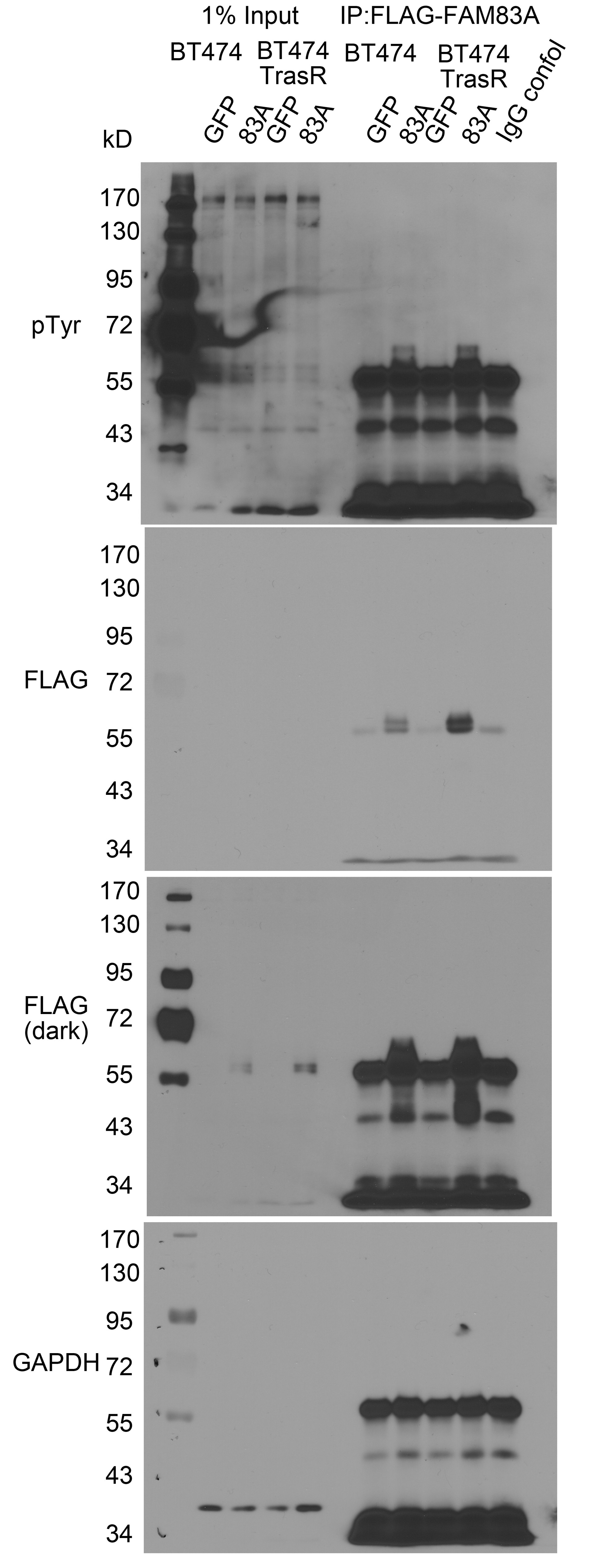

Supplement: S10 Fig — (TIF) [file pone.0176778.s010.tif]

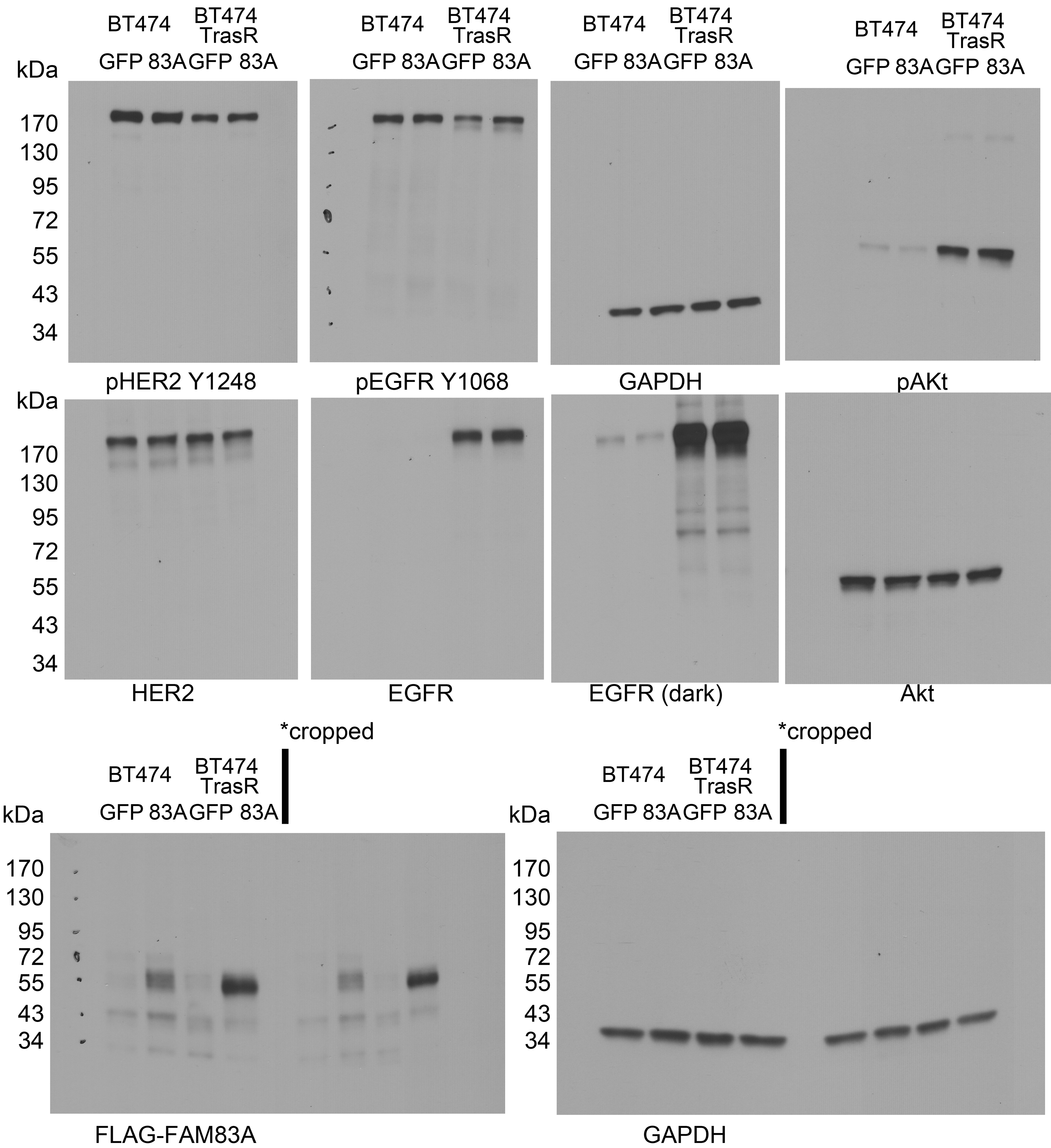

Supplement: S11 Fig — (TIF) [file pone.0176778.s011.tif]

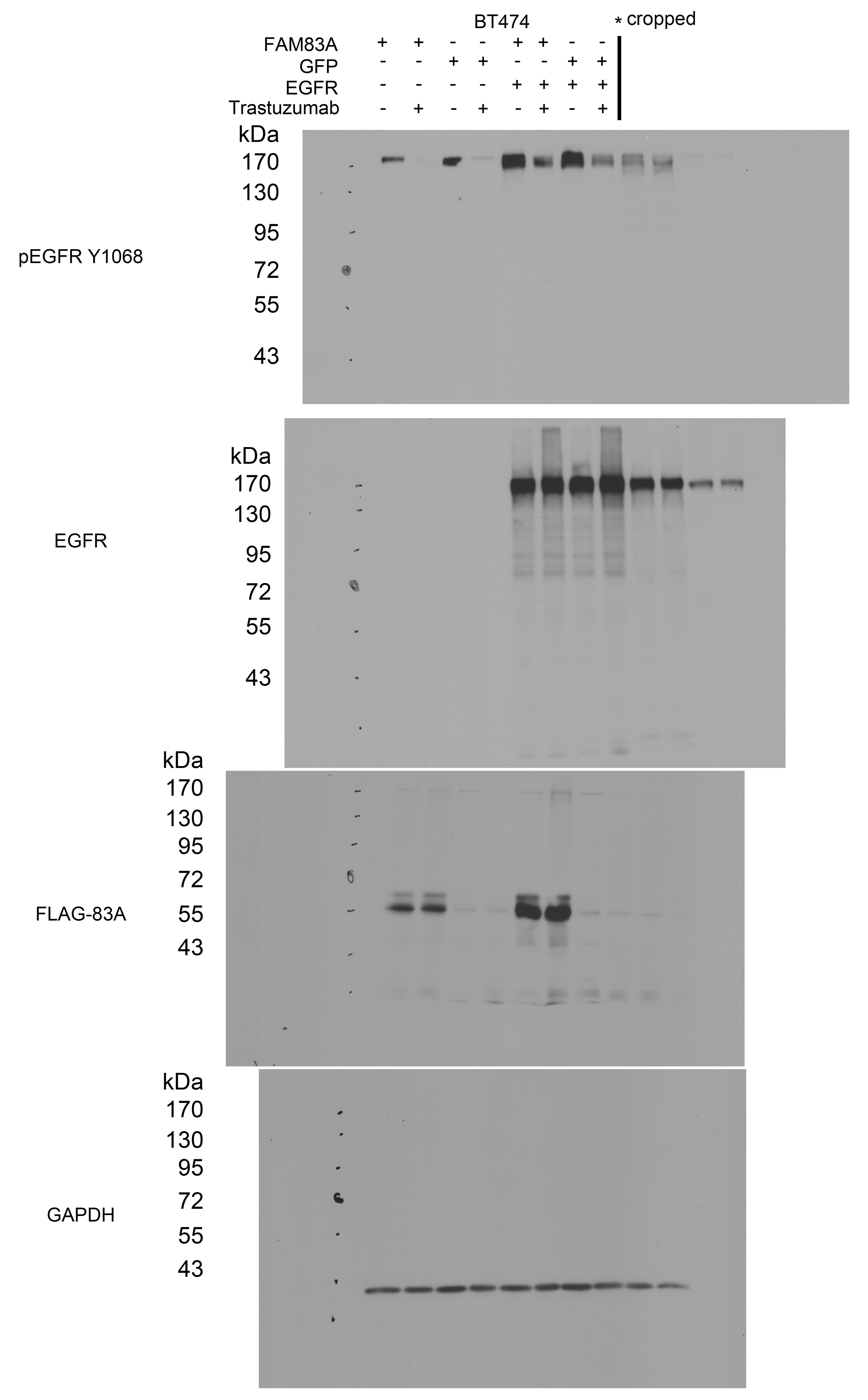

Supplement: S12 Fig — (TIF) [file pone.0176778.s012.tif]

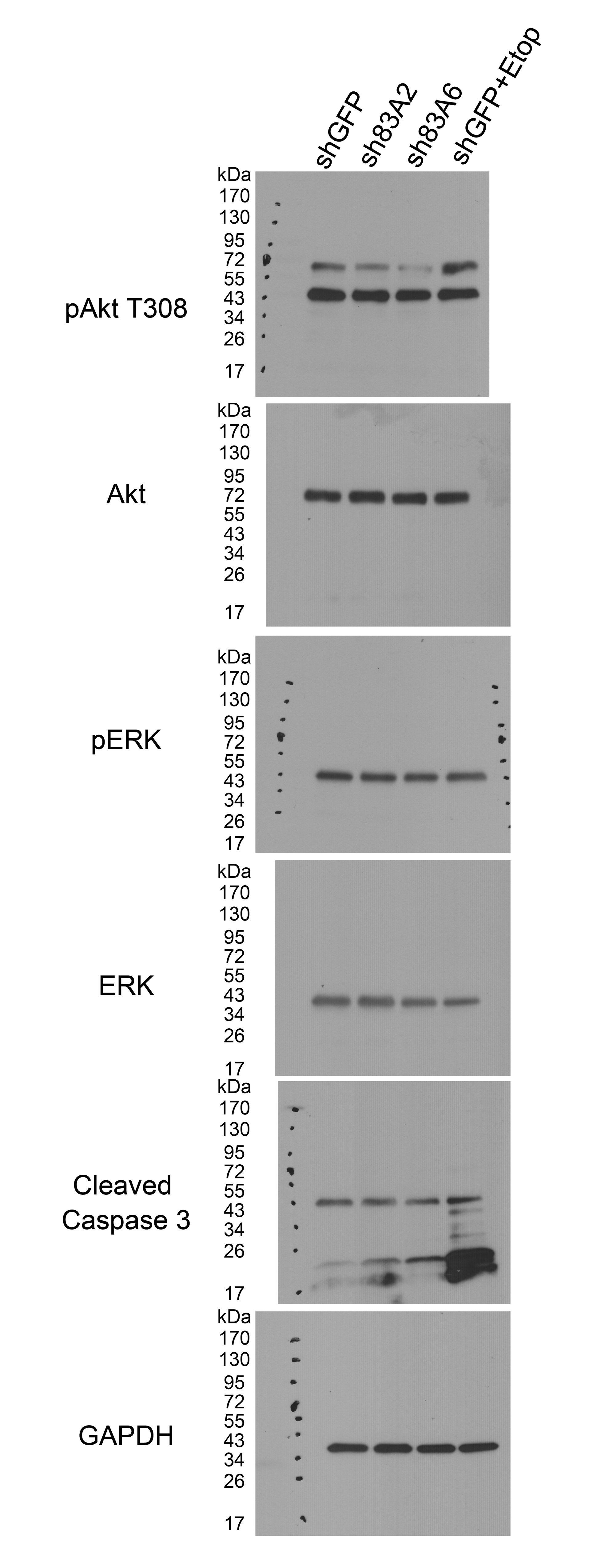

Supplement: S13 Fig — (TIF) [file pone.0176778.s013.tif]
